# Adenoviral Vectors Expressing Optimized preM/E Genes of WNV Deliver Long-Term Protection Against Lethal West Nile Virus Challenge

**DOI:** 10.3390/vaccines13121177

**Published:** 2025-11-21

**Authors:** Tatiana A. Ozharovskaia, Olga V. Zubkova, Elizaveta V. Korobova, Inna V. Dolzhikova, Denis I. Zrelkin, Olga Popova, Polina P. Goldovskaya, Anna V. Kovyrshina, Anastasia I. Korobkova, Irina A. Favorskaya, Irina V. Vavilova, Daria M. Grousova, Ilya D. Zorkov, Anna A. Iliukhina, Irina A. Ermolova, Amir I. Tukhvatulin, Dmitry N. Shcherbinin, Ekaterina I. Ermolova, Marina S. Kunda, Natalia N. Ryzhova, Olga L. Voronina, Alexander S. Semikhin, Dmitry V. Shcheblyakov, Denis Y. Logunov, Alexander L. Gintsburg

**Affiliations:** 1 N. F. Gamaleya Federal Research Center for Epidemiology & Microbiology, Ministry of Health, Moscow 123098, Russiakovyrshina@gamaleya.org (A.V.K.); amir_tukhvatulin@gamaleya.org (A.I.T.); olv550@gmail.com (O.L.V.); gintsburg@gamaleya.org (A.L.G.); 2 P. Hertsen Moscow Oncology Research Institute-Branch of the National Medical Research Radiology Center, Ministry of Health of the Russian Federation, Moscow 119121, Russia; 3 Infectiology Department, I. M. Sechenov First Moscow State Medical University, Moscow 119991, Russia

**Keywords:** West Nile virus, recombinant adenoviruses, adenovirus type 2, immunogenicity, neutralizing antibodies, protective activity, cross-reactivity, flavivirus

## Abstract

**Background/Objectives**: Flaviviruses, including West Nile virus (WNV), pose global health challenges due to their worldwide distribution, pathogenicity, and lack of effective treatments or vaccines. Today, WNV is considered the most important causative agent of viral encephalitis worldwide. This study investigated the different forms of the main WNV antigen—the preM/E protein—in the context of its immunogenic and protective properties. **Methods**: The recombinant adenovirus type 2 (rAd2) vectors expressing different forms of the WNV preM/E genes were obtained using standard molecular biology techniques. Immunogenicity in mice was assessed by enzyme-linked immunosorbent assay (ELISA) and virus neutralization assay. Immunological efficacy was evaluated in a mouse viral challenge model. **Results**: The rAd2 vector expressing the West Nile virus preM/E gene with mutations in the fusion loop exhibited robust immunogenicity when administered intramuscularly either once or in a homologous prime-boost regimen. This antigen form, as part of an adenoviral vector, protected mice from death in viral challenge experiments, providing 100% survival following WNV challenge. **Conclusions**: We believe that a vaccination strategy involving a recombinant adenoviral vector based on human adenovirus type 2 and the WNV antigen represented by the preM/E gene with mutations in the fusion loop may be a promising approach for combating West Nile virus infection.

## 1. Introduction

West Nile virus (WNV) is an arbovirus that belongs to the species *Orthoflavivirus nilense*, genus *Orthoflavivirus*, family *Flaviviridae*, and is a member of the Japanese encephalitis virus complex [1]. The first isolation of WNV happened in Africa in the 1930s when K.C. Smithburn and colleagues were conducting an epidemiological study [2]. The virus was isolated from a 37-year-old woman from the West Nile District in Uganda [3].

WNV is maintained in nature through a cycle between mosquitoes (mostly *Culex* species) as vectors and birds as hosts [4]. Occasional spillover events can result in clinical disease and mortality in equines. Humans can also be affected, sometimes developing serious or fatal diseases.

The ability of WNV to spread and persist is fundamentally tied to the availability of appropriate vectors (ornithophilic mosquito species) and bird hosts that develop sufficiently high levels of viremia to infect other mosquitoes [5].

Typically, the viral load in the bloodstream of infected mammals is significantly lower than that found in birds, making it unlikely for the virus to spread to another mosquito. This is why humans and other mammals are considered ‘dead-end hosts’ [6]. Bird migration can facilitate the distribution of WNV as a natural process. Meanwhile, globalization contributes to increased contact between humans and the virus, enhancing the potential for the transport of infected mosquitoes. In addition, bird trade and human travel can further facilitate the spread of WNV [7].

WNV exhibits a relatively high level of sequence diversity. Strains isolated from different geographic regions are classified into two major lineages (lineages 1 and 2) based on nucleic acid sequence differences, along with several less prevalent lineages (lineages 3 and 4 in Russia, 5 in India, and 6 in Spain) [5,8]. Lineage 1 predominantly circulates in central and northern Africa, Europe, Australia, and the United States. Lineage 2 is endemic to southern Africa and Madagascar and was introduced into central Europe in 2005 [3]. Lineages 1 and 2 are the most relevant from a public health perspective, as they are responsible for major outbreaks in North America and Europe [9,10].

WNV is distributed worldwide; cases of the disease have been reported on all continents except Antarctica [11]. In recent years, Europe has seen both an increase in the number of cases and the severity of disease manifestations. As of 4 December 2024, 19 European countries reported 1436 confirmed cases of WNV infection (including 125 deaths) with known place of infection. The countries with the highest numbers of reported cases were Italy (455), Greece (217), Spain (138), Hungary (111), and Albania (106) [12]. In the United States, a total of 60,992 laboratory-confirmed cases of WNV infection were reported between 1999 and 2024, including 3134 associated deaths [13]. In 2023, 2628 laboratory-confirmed cases were documented, followed by a marked decrease in 2024, with 1791 cases reported. As of 9 September 2025, 771 human WNV disease cases have been reported, of which 490 were classified as neuroinvasive disease [13].

For many years, WNV did not represent a significant threat to the population of the Russian Federation. However, a sharp deterioration in the epidemiological situation occurred in 1999, during a large outbreak in the Volgograd region, where the number of laboratory-confirmed cases reached approximately 500, with a mortality rate of around 10% [14]. Since then, WNV cases have been registered annually in Russia. In 2024, 440 cases were reported, which was more than two times higher than in 2023 (210 cases) and 1.2 times above the long-term average of 174.8 cases [15,16]. In 2024, the incidence rate of West Nile virus in the Russian Federation was 0.30 per 100,000 population, which is 2.6 times higher than the long-term average of 0.11 for the period 2010–2023 [16]. The most affected areas are the southern regions of Russia, especially the Astrakhan region [14].

Warm weather is a factor that extends the lifespan of blood-feeding mosquitoes responsible for transmitting WNV. West Nile fever is primarily prevalent in countries with hot climates, and the southern regions of Russia are also classified as endemic for this infection. Climate change is expanding the geographic range of WNV-competent mosquitoes, and they are now present in central Russia and the Volga region [17].

Approximately 80% of WNV infections are asymptomatic. The remaining 20% result in West Nile fever, which typically manifests as a mild febrile illness with symptoms such as muscle pain, headache, fatigue, nausea, rash, and hyperthermia [18]. Among these symptomatic cases, the case fatality rate is around 10%, but it is significantly higher in elderly and immunocompromised individuals [19,20]. However, in approximately 1 out of every 100–150 cases, WNV infection progresses to a severe neuroinvasive disease, such as meningitis or encephalitis, which can result in paralysis or even death [21]. For example, outbreaks of WNV meningitis and encephalitis were reported in Bucharest, Romania (1996); Volgograd, Russia (1999); and Israel (2000) [11]. Today, WNV is considered the most important causative agent of viral encephalitis worldwide [11,22].

Like other flaviviruses, WNV contains a single-stranded, positive-sense RNA genome of approximately 11 kb, which encodes 10 genes flanked by 5′- and 3′-untranslated regions [11]. The genome is translated into a single polyprotein, which is co- and posttranslationally cleaved into three structural proteins—Capsid (C), pre-Membrane/Membrane (preM/M), and Envelope (E)—and seven nonstructural (NS) proteins: NS1, NS2A, NS2B, NS3, NS4A, NS4B, and NS5 [11].

The WNV virion has icosahedral symmetry and measures approximately 50 nm in diameter, lacking surface projections or spikes [23,24]. The viral particle is surrounded by a lipid bilayer and composed of C protein, which associates with the RNA genome and mediates viral assembly [11,24]. Heterodimers of preM and E proteins are embedded in the lipid bilayer and are exposed on the surface of the virion [25]. The E protein contains an ectodomain that is anchored in the viral envelope. This ectodomain includes three major domains: I, II, and III. Domain II (DII) contains the fusion loop (FL), while Domain III (DIII) is responsible for binding to the host cell receptor [26]. It has been established that most antibodies produced during flavivirus infection are directed against epitopes in the E and NS1 proteins [27,28]. However, the E protein remains the main focus of vaccine research because it elicits the production of virus-neutralizing antibodies [29,30]. Moreover, a recombinant WNV E protein has entered phase I clinical trials as a vaccine candidate [31].

Although multiple WNV vaccine candidates have been developed—including two live attenuated chimeric vaccines, first- and second-generation DNA vaccines, one recombinant subunit vaccine, and two inactivated whole-virus vaccines—only one candidate, ChimeriVax-WN02, has thus far completed phase II clinical trials [32]. ChimeriVax-WN02 is a live attenuated recombinant vaccine based on the yellow fever 17D virus backbone, in which the preM and E genes have been replaced with the corresponding genes from WNV [33]. Despite completing phase II trials, ChimeriVax-WN02 has not progressed to phase III, possibly due to concerns regarding efficacy, safety, or commercial viability.

In this study, we developed and evaluated the immunogenicity and protective activity of several adenovirus vectors expressing different variants of the WNV preM and E protein genes. Adenoviral vector-based vaccines offer a favorable balance of safety and immunogenicity, especially compared to vaccines based on replication-competent viruses such as ChimeriVax-WN02. We demonstrated that immunization of mice with any of the six adenoviral vectors expressing various WNV antigen forms induced a robust WNV-specific humoral immune response. Furthermore, the antibodies induced by our vectors did not exhibit cross-neutralizing activity against tick-borne encephalitis virus. Among the tested antigen variants, the most promising was selected based on immunogenicity data, and that one provided 100% protection against lethal infection caused by WNV lineage 2.

## 2. Materials and Methods

### 2.1. Bacterial Strains and Cell Lines

To obtain plasmid constructs, we used the laboratory strain of *Escherichia coli* DH5α (New England Biolabs, Ipswich, MA, USA) and the *E. coli* BJ5183 strain (Stratagene, La Jolla, CA, USA).

The HEK293 (human embryonic kidney) and Vero E6 (kidney of an African green monkey) cell lines were obtained from the Russian Collection of Vertebrate Cell Lines (Moscow, Russia). These cell lines were cultured in Dulbecco’s Modified Eagle Medium (HyClone, Logan, UT, USA) supplemented with 8% fetal bovine serum (HyClone, USA), 25 mL of 7.5% sodium bicarbonate (PanEco, Moscow, Russia), 146 mg/L of L-glutamine (PanEco, Moscow, Russia), and a penicillin–streptomycin mixture (PanEco, Moscow, Russia), at 37 °C in a humidified atmosphere containing 5% CO_2_.

### 2.2. Viruses

Different lineages of WNV (lineages 1, 2, and 4) were obtained from the State Virus Collection (FSBI “National Research Centre for Epidemiology and Microbiology named after the honorary academician N.F. Gamaleya”). All virus-related work was conducted under Biosafety Level 3 (BSL-3) containment conditions.

### 2.3. Generation of Recombinant Plasmids Encoding Various Forms of the Native preM/E Gene Sequence

Variants of the non-optimized WNV antigen sequences were generated using standard molecular cloning techniques. For this purpose, WNV sample No. 13H109H (lineage 1) was obtained from the State Virus Collection (FSBI “National Research Centre for Epidemiology and Microbiology named after the honorary academician N.F. Gamaleya”). Viral RNA was isolated using TRIzol™ Reagent (Thermo Fisher Scientific, Waltham, MA, USA). cDNA was synthesized from purified RNA using SuperScript™ IV Reverse Transcriptase (Thermo Fisher Scientific, USA). The resulting cDNA served as a template for PCR amplification of the preM/E gene, which was then cloned into the pAL2-T plasmid (Evrogen JSC, Moscow, Russia).

To reconstruct the native preM/E gene, three overlapping fragments were amplified from the WNV genome and assembled via overlap extension PCR. Primer sequences are listed in Table A1. The primary sequence of each gene construct was confirmed by Sanger sequencing.

Since each construct required the addition of a SEAP leader sequence and the removal of a HindIII restriction site located within the E gene, fragments were either amplified from the pAL2-T-preM-E plasmid or synthesized de novo using primers (Appendix A, Table A1). To introduce mutations into the FL region, site-directed mutagenesis by PCR was performed using the primers 922_FusDam_1, 923_FusDam_2, 924_FusDam_3, 912_anti_vector-F, and 913_anti_vector-R. All PCR reactions were performed using FastPfu polymerase (Trans, Beijing, China) for 30 cycles with an annealing temperature of 55 °C. Cloning was carried out using the Sequence and Ligation Independent Cloning (SLIC) method, with pAL2-T as the vector. Final constructs were confirmed by Sanger sequencing. The amino acid preM/E sequence matched the corresponding sequence from GenBank (accession number KU978768.1) except for one substitution (N301D).

### 2.4. Generation of Recombinant Plasmids Encoding Various Forms of the Codon-Optimized preM/E Gene

Amino acid sequences of WNV preM/E from strains belonging to genotype 1a were retrieved from the GISAID database. A consensus sequence was generated using Geneious^®^ 10.2.3 software. This sequence matched the preM/E sequence (aa. 124–791) from GenBank (accession number AHI43626.1). The sequence, named DIIIopt, contains the DIII domain and stem (amino acids 467–620 of the full-length preM/E sequence). The FDopt sequence was derived from the preM/E sequence and includes the following mutations: T265A, M266G, W290R, and L296R. A SEAP leader sequence was also added at the beginning of each gene.

Nucleotide sequences encoding different forms of the gene were optimized for mammalian expression, taking into account parameters such as the codon adaptation index, GC content, alternative splicing signals, and homopolymeric runs exceeding 10 nucleotides. The final codon-optimized sequences were synthesized by Evrogen JSC (Moscow, Russia) and delivered in an intermediate plasmid vector.

### 2.5. Construction of Recombinant Adenoviral Vectors Based on Human Adenovirus Type 2

To construct the recombinant adenovirus type 2 (rAd2) based WNV vaccine vectors, WNV antigen genes were first subcloned into the pShUni shuttle vector using standard molecular cloning techniques. As a result, six intermediate plasmids were obtained, encoding both optimized and non-optimized sequences of various forms of the WNV prM/E gene.

Once the pShUni shuttle plasmids were prepared, rAd2-based vectors were generated via homologous recombination in *E. coli*, as previously described [34]. The parental pAd2ΔE1/E3 plasmid contained deletions in the E1 and E3 regions of the adenoviral genome. Final recombinant plasmids, which included a full-length adenovirus type 2 (Ad2) genome with E1 and E3 deletions and an expression cassette encoding various forms of the WNV prM/E gene inserted into the E1 region, were analyzed by PCR, restriction mapping, and full-genome sequencing.

### 2.6. Production and Propagation of Recombinant Adenoviruses

rAd2 vectors were rescued in HEK293 cells via lipofection of linearized plasmids, as previously described [34]. Briefly, to generate the recombinant adenovirus Ad2-Allopt, the pAd2ΔE1/E3-Allopt plasmid was used. HEK293 cells were seeded in six-well plates and incubated overnight to reach approximately 80% confluency. To remove bacterial sequences, the plasmid DNA was digested with the restriction enzyme PacI and then transfected into HEK293 cells using Lipofectamine 2000 (Thermo Fisher Scientific, USA) according to the manufacturer’s instructions. After viral cytopathic effects (CPE) were observed using a CKX41 inverted microscope (Olympus, Tokyo, Japan), the cells and culture medium were subjected to three freeze–thaw cycles to release viral particles. The remaining five rAd2 vectors were generated using the same procedure.

The generated adenoviral stocks were analyzed by real-time PCR. Verified rAd2 vectors were amplified in HEK293 cells and purified by ultracentrifugation through cesium chloride gradients using standard protocols [34]. Briefly, rAd2 were purified in two stages. First, virions were concentrated by sedimentation through a step gradient of CsCl solutions (1 mL–1.375; 3 mL–1.365; 3 mL–1.355), with densities verified by refractometry. Ultracentrifugation was performed in an Optima XPN-90 (Beckman Coulter, Brea, CA, USA) with an SW41 rotor at 35,000 rpm and 14 °C for 1 h 15 min. The opalescent band at the interface of the 1.355 and 1.365 gradients, containing recombinant adenovirus, was collected. In the second stage, this material was purified by ultracentrifugation over 8 mL CsCl (1.365), at 41,000 rpm and 15 °C for 20 h in an SW41 rotor, and the resulting opalescent band in the lower third of the tube was harvested. All rAd2 vectors were titrated on HEK293 cells using a 50% Tissue Culture Infectious Dose (TCID_50_) assay [35].

The number of adenoviral particles in each preparation was quantified spectrophotometrically according to the method of Maizel et al. [36]. In this approach, an optical density of 1.0 at 260 nm corresponds to 1.1 × 10^12^ viral particles per milliliter. To release viral DNA and proteins, samples were treated with 0.1% SDS, which fully disrupts the virions. Ultraviolet absorbance of the lysed virus was then recorded at 320 nm (baseline correction), 260 nm (DNA), and 280 nm (protein). All measurements were performed in triplicate using a NanoDrop 2000c spectrophotometer (Thermo, Waltham, MA, USA).

The final virus preparations were further tested by real-time PCR to confirm the presence of the inserted target gene and the Ad2 hexon gene, and to confirm the absence of replication-competent adenoviruses. For real-time PCR, 5X qPCRmix-HS SYBR (Evrogen JSC, Moscow, Russia) was used according to the manufacturer’s instructions. Each reaction contained 100 ng of total DNA extracted with the Wizard^®^ Genomic DNA Purification Kit (Promega, Madison, WI, USA) from cells infected with recombinant adenovirus. Real-time PCR assays were performed using the CFX96 Real-Time PCR Detection System (Bio-Rad, Hercules, CA, USA) under the following cycling conditions: 95 °C for 5 min, 40 cycles of 15 s at 95 °C, 30 s at 60 °C, and 20 s at 72 °C. The primers are described in Table A2.

### 2.7. Analysis of Antigen Expression at the mRNA Level

To indirectly assess WNV gene expression by recombinant adenoviruses at the mRNA level, Vero E6 cells were seeded in 35 mm Petri dishes at a density of 1 × 10^6^ cells per dish and incubated overnight until reaching approximately 80% confluence. The cells were then infected with rAd2 vectors at a multiplicity of infection (MOI) of 0.5 TCID_50_ per cell. For the negative control (k–), 50 μL of PBS was added per dish. After 72 h, cells were harvested for RNA extraction. Total RNA was isolated using TRIzol™ reagent (Invitrogen, Waltham, MA, USA) according to the manufacturer’s protocol. To remove the DNA, RQ1 RNase-Free DNase (Promega, Madison, WI, USA) was used according to the manufacturer’s instructions. RNA concentrations were measured using a NanoDrop 2000c spectrophotometer (Thermo Fisher Scientific, Waltham, MA, USA).

For reverse transcription real-time PCR, 5× OneTube PCRmix SYBR (Evrogen JSC, Moscow, Russia) was used according to the manufacturer’s instructions. Each reaction contained 100 ng of total RNA. Amplification was carried out using the CFX96 Real-Time PCR Detection System (Bio-Rad, Hercules, CA, USA). For detection of the native WN-DIII sequence, the primers WN DIII F3 (GCAGAGATTAGCCGCCCTA) and WN DIII R3 (ATGGACCTGTCACGAGCA) were used. For the codon-optimized DIII sequence, the primers DIII opt F (GGAACAGATGGGCCTTGC) and DIII opt R (GCTCACCACGACCCACTA) were used.

### 2.8. Evaluation of WNV E Gene Expression by Western Blotting

Vero E6 cells were seeded in 35 mm Petri dishes and incubated overnight to reach 70% confluence. The cells were then infected with Ad2-All, Ad2-FD, Ad2-DIII, Ad2-Allopt, Ad2-FDopt, or Ad2-DIIIopt vectors at a dose of 10 PFU per cell. Recombinant Ad2 expressing EGFP (rAd2-EGFP) was used as a control. Expression of the WNV E protein variants was assessed 48 h post-infection by Western blotting using a rabbit polyclonal antibody specific to the WNV E protein (West Nile Virus [lineage 1, strain NY99] E/Envelope Antibody, Rabbit PAb 40345-T16, Sino Biological, Beijing, China), followed by incubation with an HRP-conjugated secondary antibody (Donkey Anti-Rabbit IgG H&L [HRP], ab6802, Abcam, Cambridge, UK).

### 2.9. Laboratory Animals

All animal procedures were conducted in strict accordance with the Russian National Standard for Good Laboratory Practice (GOST R 53434-2009) [37]. Six-week-old female BALB/c mice (18–20 g) were obtained from the Pushchino Animal Breeding Facility (Russia). The animals were housed in ISOcage systems (Tecniplast, Buguggiate, Italy) with free access to food and water.

The study was approved by the local independent ethics committee of the FSBI “National Research Centre for Epidemiology and Microbiology named after the honorary academician N.F. Gamaleya” of the Ministry of Health of the Russian Federation (protocol No. 35 dated 24 January 2023).

### 2.10. Animal Immunization and Serum Collection

To evaluate the antigen-specific humoral immune response, BALB/c mice (6 animals per group) received either a single intramuscular injection or a prime-boost regimen (second injection 21 days later) of 10^10^ viral particles (vp) of the tested adenoviral vectors in a total volume of 100 µL. The control group received an equivalent volume of PBS. Blood samples were collected from the tail vein 14, 28, and 42 days post-immunization for subsequent serum separation. The blood samples were incubated at 37 °C for 30 min and then centrifuged at 300× *g* for 10 min. The resulting sera were stored at −70 °C until further analysis.

### 2.11. Determination of WNV E-Specific Antibody Titers by ELISA

The titers of WNV E-specific antibodies in mouse serum were measured by enzyme-linked immunosorbent assay (ELISA) using a standard protocol. Recombinant WNV E protein (lineage 1, 40345-T16, Sino Biological, Beijing, China) was used as the coating antigen. Secondary horseradish peroxidase (HRP)-conjugated anti-mouse IgG antibodies (NXA931, GE Healthcare, Chicago, IL, USA) were used for detection. Optical density (OD) was measured at 450 nm using a Multiscan FC plate reader (Thermo Fisher Scientific, Waltham, MA, USA). Antibody titers were defined as the reciprocal of the highest serum dilution with an OD value at least twice that of the negative control.

### 2.12. Growth and Characterization of WNV

All work involving WNV was performed in a BSL-3 laboratory under the supervision of at least two personnel, in accordance with SanPiN 3.3686-21 ‘Sanitary and Epidemiological Requirements for the Prevention of Infectious Diseases’.

For virus stock production, suckling mice were intracerebrally infected with WNV. After 72 h, animals were euthanized, and brains were collected. Brain tissue was homogenized using glass beads and centrifuged for 10 min at 200× *g*. The supernatant was collected and stored at –80 °C. The infectious activity of the virus was assessed by infecting Vero E6 cells grown as monolayers in 96-well plates (SPL Lifesciences, Pocheon-si, Republic of Korea) with 10-fold serial dilutions. After 7 days of incubation, cytopathic effects were evaluated visually. Virus titers were calculated using the Spearman-Kärber method and expressed as log_10_ TCID_50_/_mL_. Viral identity was confirmed by real-time PCR.

### 2.13. Determination of Virus-Neutralizing Antibody (NtAb) Titers Against WNV

Sera from vaccinated mice were heat-inactivated at 56 °C for 30 min. For the virus neutralization assay, sera were serially two-fold diluted from 1:10 in DMEM medium (HiMedia, Mumbai, India) supplemented with 2% fetal bovine serum (Capricorn, Düsseldorf, Germany), and tested in duplicate in 96-well culture plates. Next, 50 μL of WNV (2000 TCID_50_/_mL_) was added to each well and incubated at 37 °C with 5% CO_2_ for 1 h. The virus-serum mixtures were then transferred onto Vero E6 cells. Plates were incubated for 7 days at 37 °C with 5% CO_2_. The NtAb titer was defined as the highest serum dilution that inhibited WNV replication in 50% of wells compared to the virus-only control.

### 2.14. Cloning and Expression of Tick-Borne Encephalitis Virus (TBEV) E Proteins

The ectodomains of the TBEV E protein from the Far-Eastern (Sofjin strain) and Siberian (Vologda-2 strain) subtypes were produced using a mammalian expression system. The amino acid sequence of the E protein ectodomain from the Far-Eastern subtype was almost identical to the sequence in GenBank (accession number ACO82048.1; aa 284–683), except for four amino acid substitutions: H413Y, R454K, Q543H, and T646I. The amino acid sequence of the E protein ectodomain from the Siberian subtype matched the GenBank sequence (accession number QPD02203.1; aa 261–680), except for two substitutions: I408T and Y535F.

The DNA sequences encoding the E protein ectodomains, comprising three domains (DI, DII, and DIII), were fused at the N-terminus with the signal peptide of the human IgG1 heavy chain (MGWSLILLFLVAVTRVLS) and at the C-terminus with a 10 × His tag. These constructs were cloned into the pCEP4 vector. Recombinant plasmids were transiently transfected into HEK293 cells using linear polyethylenimine (PEI). E proteins were purified by immobilized metal affinity chromatography (IMAC) using an ÄKTA Start system (Cytiva, Marlborough, MA, USA) and a HisTrap column (Cytiva, Marlborough, MA, USA) from culture supernatants collected on days 5 to 6 post-transfection.

### 2.15. Determination of TBEV E-Specific Antibody Titers by ELISA

The titers of TBEV E-specific antibodies in mouse serum were measured by ELISA using a standard protocol. TBE virus E proteins (Sofjin and Vologda-2 strains) were used as coating antigens. Secondary HRP-conjugated anti-mouse IgG antibodies (A9044-2ML, Sigma-Aldrich, St. Louis, MO, USA) were used for detection. OD was measured at 450 nm using a Multiscan FC plate reader (Thermo Fisher Scientific, Waltham, MA, USA). Antibody titers were defined as the reciprocal of the highest serum dilution with an OD value at least twice that of the negative control.

### 2.16. WNV Challenge

To ensure that WNV would cause a lethal infection in mice, immunosuppressive therapy was administered. Immunosuppression was induced using dexamethasone and cyclophosphamide according to the following regimen: dexamethasone was administered intraperitoneally at a dose of 10 mg/kg daily for 7 days, and cyclophosphamide was administered intraperitoneally at a dose of 150 mg/kg three days prior to infection. The control group consisted of uninfected animals with induced immunosuppression.

BALB/c mice with induced immunosuppression (n = 5 per group) were challenged intraperitoneally with 6 log_10_ TCID_50_ of WNV lineage 2. Following infection, animals were observed daily for 35 days to monitor body weight changes and survival. Animals that lost more than 25% of their initial body weight were humanely euthanized in accordance with ethical guidelines. At the end of the observation period, all surviving animals were euthanized.

### 2.17. Statistical Analysis

Statistical analysis was performed using GraphPad Prism 8.0.1 (GraphPad Software, San Diego, CA, USA) and Microsoft Excel (Microsoft, Redmond, WA, USA). The Mann–Whitney U test was used for comparisons between independent groups. Differences were considered statistically significant at *p* < 0.05.

## 3. Results

### 3.1. Generation of rAd2 Vectors Expressing Different Variants of WNV M and E Protein Genes

At the initial stage of this study, specific antigenic sequences of the WNV were selected. We focused on the gene encoding the precursor of the M and E proteins (preM/E), either in its wild-type form or with a mutation introduced into the fusion loop of the E protein. These mutations were introduced to reduce the risk of antibody-dependent enhancement (ADE) of infection. The E protein was chosen as a target due to its critical role in eliciting virus-neutralizing antibodies that contribute to protective immunity. Given that the pre-membrane (preM) protein of WNV functions as a chaperone to ensure proper folding of the E protein [38,39], the preM/E gene was included in the study. Along with the full-length preM/E protein, domain III (DIII) of the E protein was also selected for investigation. The wild-type preM/E gene sequence was derived from a WNV strain belonging to lineage 1. The other three variants were based on a codon-optimized consensus sequence, also corresponding to lineage 1.

To construct rAd2-based WNV vectors, different versions of the WNV antigen genes were subcloned into our shuttle plasmids. For the non-optimized variants of the preM/E gene and the DIII domain sequence, the corresponding genome fragments were amplified using high-fidelity PCR and initially cloned into an intermediate vector (pAL2-T, Evrogen JSC, Russia). These were subsequently subcloned into the shuttle plasmids used for recombination in *E. coli*. The optimized variants of the preM/E gene and DIII domain were chemically synthesized and delivered in intermediate plasmid form. These genes were also subcloned into the shuttle vectors for further recombination in *E. coli*. All final constructs were verified by sequencing.

**Figure 1:**
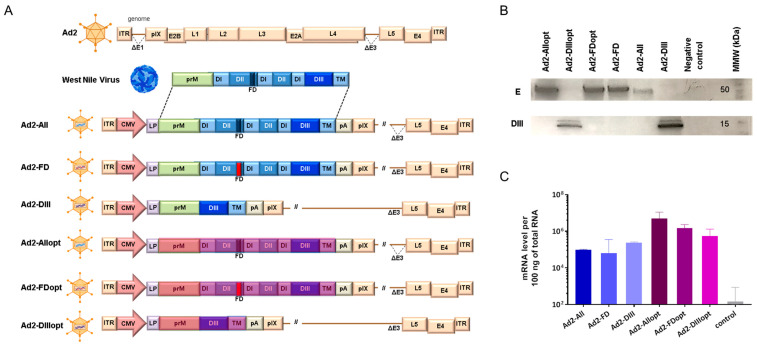
Construction and characterization of rAd2 vectors expressing different variants of the WNV preM/E genes. (**A**) Schematic representation of the recombinant adenoviruses obtained in this study. (**B**) Western blot analysis of Vero E6 cell lysates from rAd2-infected cells. (**C**) Antigen expression of rAd2 was assessed at the mRNA level. The graph shows the mean expression levels of the E protein gene along with the standard deviation. All pairwise comparisons among rAd2 groups, and between each rAd2 group and the non-transduced control, were statistically significant (*p* < 0.05; Mann–Whitney U test). The original Western blot figures can be found in Supplementary File S1.

Once the shuttle plasmids were assembled, the rAd2 vectors expressing genes of the WNV proteins were generated as previously described [34]. The rAd2 genome was based on a modified Ad2 backbone containing deletions in the E1 and E3 regions. The structural organization of the constructed WNV vectors is illustrated in Figure 1A. The identity of the generated rAd2 constructs was further confirmed by real-time PCR targeting the Ad2 hexon gene and the WNV gene, as well as by whole-genome sequencing.

The expression levels of different prM/E gene variants were assessed at both the protein level, using Western blot analysis (Figure 1B), and the mRNA level (Figure 1C), using reverse transcription real-time PCR. Western blot analysis revealed that cells transduced with adenoviral vectors carrying the full-length WNV E glycoprotein gene (Ad2-All, Ad2-FD, Ad2-Allopt, and Ad2-FDopt) produced a glycoprotein E band of approximately 50 kDa, consistent with the predicted molecular weight (44 kDa, with apparent increase likely due to glycosylation). In cells transduced with vectors encoding the DIII domain of the E protein (Ad2-DIII and Ad2-DIIIopt), a specific band corresponding to the DIII protein (~15 kDa) was detected, matching the calculated molecular weight of 13.5 kDa. These results demonstrate that all six constructed adenoviral vectors expressing various forms of the WNV M and E protein genes are functionally active, as confirmed by the detection of the E protein in transduced cells via Western blot.

mRNA expression of the WNV E protein gene was detected in all cell samples transduced with Ad2-All, Ad2-FD, Ad2-DIII, Ad2-Allopt, Ad2-FDopt, and Ad2-DIIIopt vectors. Notably, expression levels were higher in cells transduced with rAd2 vectors expressing the optimized variants of the gene, with the highest level observed in cells transduced with Ad2-Allopt. Based on these findings, the optimized antigen variants (Ad2-Allopt, Ad2-FDopt, and Ad2-DIIIopt) were selected for further investigation.

### 3.2. rAd2 Vectors Expressing Different WNV preM/E Gene Variants Induce a Humoral Immune Response in Mice

#### 3.2.1. Study of the Immunogenicity of Different Variants of the Optimized WNV preM/E Protein Gene After Single and Double Administration

**Figure 2:**
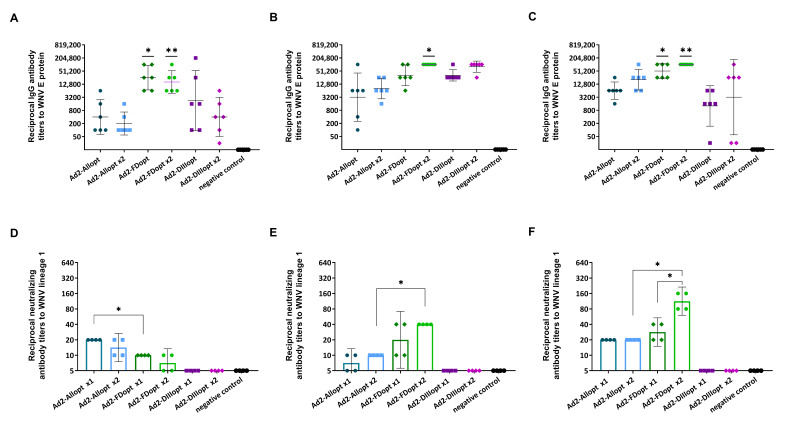
Immunogenicity of rAd2 vectors expressing different WNV preM/E gene variants. Scatter plots show geometric mean titers (GMTs) and 95% confidence intervals (CIs) for each group (n = 6 mice/group). (**A**–**C**) WNV E-specific IgG titers in the blood serum of immunized animals: (**A**) Day 14. * *p* < 0.05, comparison of Ad2-FDopt 1x with other forms (except for Ad2-DIIIopt x1 and Ad2-FDopt 2x); ** *p* < 0.05, comparison of Ad2-FDopt 2x with other forms (except for Ad2-DIIIopt x1 and Ad2-FDopt 2x). (**B**) Day 28. * *p* < 0.05, comparison of Ad2-FDopt 2x with other groups (except for Ad2-DIIIopt x2 and Ad2-FDopt 1x). (**C**) Day 42. * *p* < 0.05, comparison of Ad2-FDopt 1x with Ad2-Allopt 1x and Ad2-DIIIopt 1x; ** *p* < 0.05 comparison of Ad2-FDopt 2x with other groups, except for Ad2-FDopt 1x. (**D**–**F**) NtAb titers against WNV measured on: (**D**) day 14, (**E**) day 28 and (**F**) day 42 post-second immunization. Sera were pooled (2 mice per pool) and analyzed in duplicates.

To compare the immunogenicity of the recombinant adenoviral vectors following single or double administration, BALB/c mice were immunized intramuscularly with Ad2-Allopt, Ad2-DIIIopt, or Ad2-FDopt at a dose of 1 × 10^10^ vp per mouse. Blood samples were collected on day 42 after the first immunization to determine titers of WNV lineage 1 E-specific IgG antibodies (Figure 2A–C) and neutralizing antibodies (NtAbs) (Figure 2D–F). No WNV E-specific IgG antibodies were detected in serum samples from the control group. Interestingly, for all three time points analyzed (days 14, 28, and 42), no significant differences in IgG titers were observed between single and double immunizations with the same antigen variant. However, on day 14, IgG titers induced by Ad2-FDopt were significantly higher (*p* < 0.05) compared to other groups, with the exception of the single DIII immunization group. On day 28, IgG titers in the group that received two doses of Ad2-FDopt were also significantly higher (*p* < 0.05), except when compared to the group receiving two doses of Ad2-DIIIopt.

The highest IgG titers on day 42 were observed in the group immunized with Ad2-FDopt both once (GMT: 51,200) and twice (GMT: 102,400). The titers following single immunization with Ad2-FDopt were significantly higher (*p* < 0.05) than those following single immunization with Ad2-Allopt and Ad2-DIIIopt. After two immunizations, Ad2-FDopt elicited significantly higher IgG titers compared to all other groups (*p* < 0.05).

Neutralizing antibody titers increased over time, from day 14 to day 42 (Figure 2D–F). The highest levels were observed in mice immunized twice with Ad2-FDopt, particularly on days 28 and 42 after the second immunization. No NtAbs were detected in any group immunized with Ad2-DIIIopt, regardless of the number of doses administered.

#### 3.2.2. Cross-Reactivity of Neutralizing Antibodies Against Different WNV Lineages

**Figure 3:**
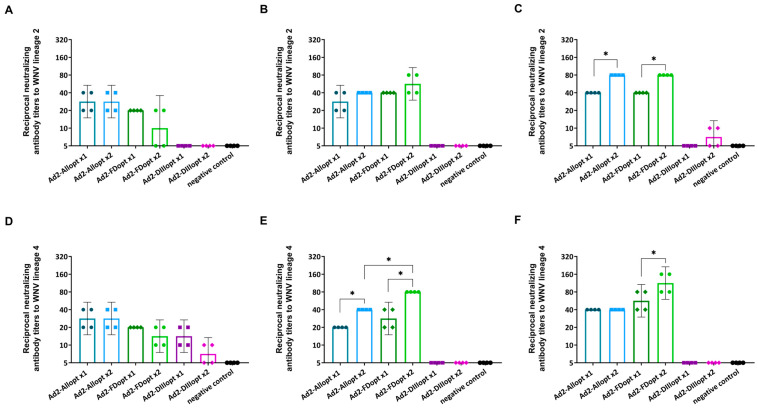
Analysis of cross-reactivity of neutralizing antibodies in mice immunized with rAd2 expressing different variants of the optimized WNV preM/E genes against WNV lineages 2 (**A**–**C**) and 4 (**D**–**F**). Scatter plots show the geometric mean titer (GMT) and 95% confidence interval (CI) for each group (n = 6 mice/group); sera were pooled (two mice per pool) and analyzed in duplicate. The graphs represent NtAb titers against WNV on days 14 (**A**,**D**), 28 (**B**,**E**), and 42 (**C**,**F**) after the second immunization. * *p* < 0.05.

To assess cross-reactivity, sera from mice immunized once or twice with Ad2-Allopt, Ad2-DIIIopt, or Ad2-FDopt were tested for neutralizing activity against WNV lineages 2 and 4 in addition to lineage 1. Results are presented in Figure 3.

Similar to lineage 1, a time-dependent increase in NtAb titers was observed for lineages 2 and 4 (Figure 3). NtAbs were detected in the Ad2-Allopt and Ad2-FDopt groups (both single and double immunizations), whereas little to no virus-neutralizing activity was detected in the Ad2-DIIIopt group, except for low levels against lineage 2 on day 42 and lineage 4 on day 14 following double immunization. Statistical comparison of NtAb titers against lineage 2 on days 14 and 28 revealed no significant differences between the Ad2-Allopt, Ad2-DIIIopt, and Ad2-FDopt groups, indicating that full-length and fusion-loop-mutated antigens induce similar levels of cross-neutralizing antibodies. However, by day 42, significantly higher NtAb titers were detected in the groups immunized twice with Ad2-Allopt or Ad2-FDopt (*p* < 0.05).

For lineage 4, no statistically significant differences were found on day 14. On day 28, significantly higher NtAb titers were observed in mice immunized twice with Ad2-Allopt or Ad2-FDopt compared to single-shot immunization, with double immunization using Ad2-FDopt showing superior efficacy. On day 42, significant differences were observed only between groups immunized with Ad2-FDopt once or twice, with two doses yielding a stronger response.

#### 3.2.3. rAd2s Expressing Different WNV preM/E Gene Variants Do Not Induce Cross-Reactive Immunity Against Tick-Borne Encephalitis Virus

Mouse sera from the previous experiment (from mice that received double immunization with rAd2) were analyzed for the presence of IgG antibodies specific to the E protein of TBEV, strains Sofjin and Vologda-2. The Sofjin strain was used as it represents one of the earliest isolates and serves as the reference strain for TBEV [40]. The results showed that neither the variant containing mutations in the FL nor the full-length M and E protein variants induced cross-reactive antibodies in the immunized mice (all OD values did not exceed those of the negative control).

### 3.3. A Double Immunization with rAd2 Expressing the Optimized WNV preM/E Gene Carrying a Fusion Loop Mutation in the E Protein Protects Against Lethal WNV Infection

To assess long-term protective efficacy, we examined whether the candidate vaccine could provide sustained protection over an extended period—for example, if immunization occurs in March, whether protection persists until September. To this end, we evaluated the efficacy of recombinant adenoviral vectors Ad2-Allopt, Ad2-FDopt, and Ad2-DIIIopt against lethal WNV challenge. BALB/c mice were immunized twice (on days 0 and 28) with one of the rAd2 or PBS (control group), and then were subjected to immunosuppressive therapy with dexamethasone and cyclophosphamide and finally challenged with a lethal dose of WNV lineage 2 (6 log_10_ TCID_50_) seven months later.

**Figure 4:**
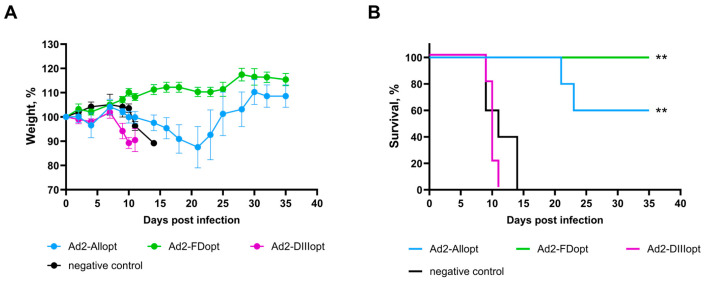
In vivo protective efficacy of Ad2-Allopt, Ad2-FDopt and Ad2-DIIIopt in mice. BALB/c mice (n = 5 per group) were immunized twice and then challenged with a lethal dose of WNV seven months later. (**A**) Body weight curves are presented as mean values ± standard error of the mean (SEM). (**B**) Survival rates of mice. The significance of survival differences was calculated using the log-rank (Mantel–Cox) test: **, *p* < 0.01.

Remarkably, all mice immunized with Ad2-FDopt survived the lethal challenge (Figure 4), showing no significant weight loss. In contrast, none of the mice immunized with Ad2-DIIIopt or PBS survived the challenge with no significant differences between these two groups. Mice immunized with Ad2-Allopt showed moderate protection, with 60% survival. One out of five mice experienced a 22% weight loss following infection. Recovery in this group began after day 21 post-challenge.

Taken together, these findings support the conclusion that the optimized preM/E antigen with a modified FL (Ad2-FDopt) is superior in terms of both immunogenicity and long-term protective efficacy. The absence of detectable morbidity and the full protection observed even several months after immunization underline the potential of this candidate for use in a long-lasting WNV vaccine.

## 4. Discussion

With the continuous emergence of novel viruses and highly pathogenic variants of known pathogens, the need for flexible, rapid-response vaccine platforms has become increasingly evident. This urgency is further heightened by global trends such as climate change, urbanization, globalization, and intensified human migration, all of which contribute to the accelerated spread of infectious diseases. The COVID-19 pandemic underscored the critical importance of preparedness and the availability of well-characterized vaccine platforms capable of rapid deployment in response to emerging threats. Among the most promising and extensively studied approaches are recombinant adenoviral vector-based platforms, which have demonstrated strong immunogenicity—eliciting both humoral and cellular responses—alongside a favorable safety profile. Their efficacy has been validated in numerous clinical trials, including those targeting SARS-CoV-2 [41,42,43], highlighting their potential as a universal solution for combating both emerging and re-emerging infections. Importantly, these platforms are particularly suitable for protecting vulnerable populations, including children, the elderly, and immunocompromised individuals.

In the context of arboviruses such as Zika virus (ZIKV), Chikungunya, and WNV, adenoviral vector-based vaccines represent a timely and rational strategy. WNV, like other members of the *Flaviviridae* family, exhibits a capacity for rapid adaptation, increased virulence, and expanding geographic distribution [44]. Over the past few decades, WNV has been responsible for multiple outbreaks across diverse regions, including Europe and North America [8]. Given the high likelihood that additional arboviruses will emerge or expand into new areas, the development of universal, platform-based vaccine solutions that offer both efficacy and broad demographic applicability is not only timely but essential.

A comprehensive understanding of immune responses to various viral antigens is crucial for the rational design of next-generation vaccines. Flaviviruses share high genetic and structural homology, particularly within the E protein, which contains conserved epitopes that can induce cross-reactive antibodies [45]. This cross-reactivity presents a considerable challenge in flavivirus vaccine development, as it may lead to ADE of infection. ADE is a phenomenon whereby non-neutralizing or sub-neutralizing antibodies, typically generated during a primary flavivirus infection, facilitate the entry of a heterologous flavivirus into Fcγ receptor-bearing cells, such as monocytes. This process enhances viral replication and has been linked to increased disease severity. ADE poses a major concern in regions where related flaviviruses circulate together and has been clinically documented in cases involving different DENV serotypes, as well as in subsequent infections involving ZIKV and DENV [46,47]. Moreover, preclinical evidence indicates that WNV antibodies can enhance ZIKV infections [48].

Given that many cross-reactive antibodies target the highly conserved FL of the E protein [45], we introduced mutations in this region to reduce ADE risk. Previous studies have demonstrated that FL-deleted or mutated ZIKV virus-like particles (VLPs) reduce ADE induction in animal models [49,50]. Following this rationale, we used WNV preM/E variants with FL mutations, hypothesizing that this would minimize cross-reactivity and enhance safety.

We selected the E protein for this study due to its role as a primary target of virus-neutralizing antibodies essential for protective immunity. In addition to the full-length E protein, we also investigated DIII, which is known to induce potent and specific antibody responses. Our findings indicate that both the mutated FL variant and the full-length M and E protein constructs did not induce cross-reactive antibodies to TBEV in mice. Future studies should examine cross-reactivity with other flaviviruses, such as DENV.

To ensure the relevance of our study, we used a wild-type preM/E gene sequence derived from a lineage 1 WNV strain, as this lineage is widely distributed and clinically significant. Understanding the genetics and immunological responses associated with this lineage is vital for developing effective countermeasures against the virus.

Additionally, three optimized variants were developed based on a consensus sequence from lineage 1. These codon-optimized constructs allowed us to evaluate the influence of nucleotide-level changes on antigen expression and immunogenicity. Although codon optimization only minimally altered the codon adaptation index (increasing it from 0.75 to 0.76), it significantly reduced the number of nucleotide repeats (>10 nt), potentially improving mRNA stability [51]. The overall sequence identity between the native and optimized genes was 77.08%.

Codon optimization significantly increased mRNA expression, particularly for the Ad2-Allopt construct, although all antigens were translated into proteins of the expected size. Taken together, these results confirm the successful generation of adenoviral vectors. On one hand, our data suggest that codon optimization alone is sufficient to enhance antigen expression in mammalian cells. On the other hand, the modest nature of the optimization may account for the relatively small differences in immunogenicity, despite the observed variation in mRNA expression levels between optimized and non-optimized constructs.

In addition to antigen selection, the choice of vector platform represents a critical consideration in vaccine development. Currently, approved vaccines against mosquito-borne flaviviruses exist only for DENV, Japanese encephalitis virus, and yellow fever virus [52], and even these face challenges such as safety concerns and complex production processes. Platforms based on mRNA or recombinant viral vectors have emerged as promising alternatives due to their rapid development, scalability and ability to induce robust immune responses.

We selected human adenovirus type 2 (Ad2) as our vector platform as it successfully combines the safety of a recombinant viral vector with efficacy. It is well established that the target gene is expressed by adenoviral vectors in the cell at a high level, which leads to strong immunogenicity. Ad2 has also been explored as a vector for ZIKV, another member of the *Flaviviridae* family, demonstrating its versatility and promise as a vaccine platform [53]. Supporting this, a recent study by Chang et al. showed that a bivalent adenovirus-vector vaccine targeting West Nile virus and Chikungunya virus elicited robust neutralizing antibody and T-cell responses in mice after a single dose, further highlighting the potential of adenoviral platforms for rapid and effective protection against multiple arboviral pathogens [54].

Given the lack of a clearly defined dominant protective antigen for WNV, we compared six different antigen variants. Our results demonstrate that the most immunogenic variant was the codon-optimized consensus preM/E construct with FL mutations. This construct induced the highest levels of WNV-specific IgG and NtAbs. Importantly, sera from immunized mice neutralized WNV strains from three different lineages, indicating broad-spectrum protection.

Despite promising results, our study has limitations. First, we did not assess T-cell responses, which are crucial for long-term immunity and viral clearance. Second, we focused exclusively on preM/E antigens and did not evaluate the immunogenic potential of NS proteins. Several studies have highlighted the role of conserved NS proteins in eliciting robust cellular immune responses [55,56]. Including both structural and non-structural antigens in future vaccine candidates may provide broader protection against WNV and more durable immunity. Assessing cellular immunity and exploring constructs that incorporate both structural and NS antigens will be important objectives for our future studies.

Recent studies have provided growing evidence that WNV can overwinter in mosquito populations in several parts of Europe, marking a concerning shift in its transmission dynamics [57]. For example, in the Czech Republic, lineage 2 WNV RNA was detected in hibernating *Culex pipiens* mosquitoes in early spring (2017), supporting the possibility of winter persistence in mosquitoes [58]. Similarly, in Germany, one pool of *Culex pipiens* collected from a hibernaculum in March 2021 tested positive for WNV RNA, closely matching previously circulating strains, which suggests local overwintering rather than reintroduction [59]. Although not all studies detect the virus in overwintering mosquitoes [60], these findings suggest that WNV may employ multiple persistence strategies, including vertical transmission in mosquitoes or low-level circulation in resident birds, to survive unfavorable conditions.

This adaptation significantly complicates traditional vector-control measures, which have largely focused on limiting transmission during peak mosquito activity in the summer and early autumn. The prospect of year-round viral maintenance raises several public health concerns: earlier seasonal outbreaks, prolonged transmission periods, and increased opportunities for spillover to humans and animals. The evidence of overwintering strengthens the case for preemptive vaccination strategies as part of integrated disease control programs.

In this study, we aimed to evaluate the long-term protective efficacy of three recombinant adenoviral vectors (Ad2-Allopt, Ad2-FDopt, and Ad2-DIIIopt) against a lethal WNV challenge. The goal was to assess whether the immunity induced by the vaccine candidates would persist over an extended period, which, in our case, was seven months. This time frame simulates a real-world scenario, such as immunization in early spring and exposure during the peak of mosquito season in late summer or early autumn. To investigate this, BALB/c mice were immunized twice with one rAd2 or PBS (as a control group), and seven months later, they were challenged with a lethal dose of WNV lineage 2.

The results demonstrated that Ad2-FDopt, a vector expressing a codon-optimized WNV preM/E gene with a mutation in the fusion loop of the E protein, provided complete protection, with 100% survival of immunized mice and no significant weight loss observed throughout the 35-day post-challenge period. These findings suggest that this construct induces a durable and effective immune response capable of neutralizing WNV infection long after vaccination.

In contrast, immunization with Ad2-DIIIopt, which expresses only the sequence of domain III of the E protein, did not confer protection, with all animals succumbing to infection by day 11. The immunogenicity of Ad2-DIIIopt in our study appears to have been insufficient to confer long-term protection, resulting in early mortality. This outcome underscores the limited protective capacity of the DIII domain alone, despite its recognized immunogenic potential. These results are somewhat unexpected, as previous studies have shown that DIII-based vaccine candidates were immunogenic, elicited high titers of WNV-neutralizing antibodies [61], and provided protection against lethal WNV challenge in mice [62].

Interestingly, Ad2-Allopt, which expresses the optimized full-length preM/E gene, conferred partial protection. Although all mice in this group exhibited substantial weight loss, 60% survived the challenge. These mice began to recover after day 21 post-challenge, which suggests that although the initial immune response was not sufficient to prevent viral replication entirely, it may have been strong enough to reduce the severity of the infection, allowing for partial recovery. This indicates that Ad2-Allopt could provide partial protection and might help reduce the overall disease burden.

In conclusion, the rAd2 vector expressing a mutated WNV preM/E antigen (with FL modifications) demonstrates strong immunogenicity following single or double intramuscular administration. This vaccine candidate provided complete protection in a lethal challenge model, with 100% survival in immunized mice. These findings suggest that this vaccination strategy holds promise for preventing West Nile virus infection.

## 5. Conclusions

Overall, the results of this study highlight the potential of the rAd2-based vector expressing the optimized WNV preM/E gene with mutations in the FL (Ad2-FDopt) for WNV vaccine development. This vaccine candidate demonstrates strong immunogenicity following single or double intramuscular administration. Also, this vector provided long-term protection in a lethal challenge model, with 100% survival in immunized mice. The complete survival and minimal weight loss observed in the Ad2-FDopt group suggest that this vaccine formulation provides strong and sustained immunity. However, the lack of protection observed in Ad2-Allopt and Ad2-DIIIopt suggests that these variants may require further optimization to improve their long-term efficacy. These findings underscore the importance of optimizing vaccine candidates to achieve both strong initial immunity and long-lasting protection. Future studies should further explore the mechanisms underlying these differences in vaccine efficacy and investigate additional strategies to enhance immune responses, including the use of adjuvants or alternative delivery methods.

## 6. Patents

An application (No. W24088918) for a Russian patent was filed on 27 December 2024.

## Data Availability

The original contributions presented in this study are included in the article/Supplementary Material. Further inquiries can be directed to the corresponding authors.

## Supplementary Materials

The following supporting information can be downloaded at: https://www.mdpi.com/article/10.3390/vaccines13121177/s1, File S1: The original Western blot figures.

## Abbreviations

The following abbreviations are used in this manuscript:

Ad2	adenovirus type 2
ADE	antibody-dependent enhancement of infection
CI	95% confidence interval
DENV	Dengue virus
E	envelope
ELISA	enzyme-linked immunosorbent assay
FL	fusion loop
GMT	geometric mean titer
HRP	horseradish peroxidase
NS	nonstructural
NtAbs	neutralizing antibodies
rAd2	recombinant adenovirus type 2
TBEV	tick-borne encephalitis virus
TCID_50_	50% Tissue Culture Infectious Dose
vp	virus particles
WNV	West Nile Virus
ZIKV	Zika virus

## Appendix A

**Table A1:** Primer sequences used to obtain non-optimized sequences of WNV preM/E gene.

Primer Name	5′→3′–Sequence
WNV_L2-F1	GTGACCCTCTCGAACTTCCAGGGCAAAG
WNV_L2-F2	TCAACGAGAGCCGCGTGTCC
WNV_L2-F3	GGCCGGAGCGATTCCTGTTGA
WNV_L2-R1	AACGAAGGCGGGGTCAGCTC
WNV_L2-R2	TCCATGGCCAGTGTCAGCGG
WNV_L2-R3	AGCATGGACGTTGACCGAAAGGAAG
553_Lider-F1	GCCGCCACCATGCTCGGCCCCTGTATGCTACTGTTGCTCTTGCTTCTGGGACTGAGACTGCAGCTTTCCCTCGGA
560_WNV-R	AGCRTGCACGTTCACGGAGAG
561_WNV-F	GTTACCCTCTCTAACTTCCAAGGGAAGG
912_anti_vector-F	CCTTTGAGTGAGCTGATACCGCTCAGATCTAAGCTTGCCGCCACCATGCTCGGCCCCT
913_anti_vector-R	ACATTTCCCCGAAAAGTGCCACCCGTCTAGAAGATATCTTATCAAGCGTGCACGTTCACGGAGAG
920_DIII_only_1	ACAACCTACGGCGTCTGTTC
921_DIII_only_2	GAACAGACGCCGTAGGTTGTTCCGAGGGAAAGCTGCAGTC
922_FusDam_1	CAAAAGCTGGGTCAGCACGTTTGTCATTGTGAGCTTCTCCGCCGGCCGGGCACGCAGCTTTGGTGG
923_FusDam_2	ACCCCTGTCCACTACTCCTTGTTTACACACAAAAGCTGGGTCAGCACGT
924_FusDam_3	CAAGGAGTAGTGGACAGGGGTCGGGGCAACGGCTGTGGACGGTTTGGTAAAGGAAGCATTGACAC
925_preME-1	CCTTGGAAGTTAGAGAGGGTAACTCCGAGGGAAAGCTGCAGTC
926_preME-2	GCCTGATGCAGAGCTCCCTC
927_preME-3	GAGGGAGCTCTGCATCAGGCTTTGGCTGGAGCCATTCCTGTG
928_Vector_F	GGTGGCACTTTTCGGGGAAATGT
929_Vector_R	GAGCGGTATCAGCTCACTCAAAGG

**Table A2:** Primers used to verify the authenticity of the obtained recombinant adenoviruses.

Primer Name	Target Sequence	5′→3′–Sequence
Ad2-hex-F	hexon gene of Ad2	CTCGAGATCAGGCTACTAAGA
Ad2-hex-R		TGTGTTTCTGCATTGTCTGAT
WN-All-opt-f	codon-optimized full-length preM/E protein gene	GCCGCATGTCCCACGATG
WN-All-opt-r		GCCCGCACCCATTGCCC
DIII opt F	codon-optimized DIII sequence	GGAACAGATGGGCCTTGC
DIII opt R		GCTCACCACGACCCACTA
WN-FD-opt-f	codon-optimized preM/E gene with mutations in the fusion loop	CCGCATGTCCCGCTGGT
WN-FD-opt-r		TCCCGCACCCATTGCCTCT
E-125R	E1 region of adenovirus type 5 genome	TTTCCCACCCTTAAGCCACG
E1b-F		GATGTGACCGAGGAGCTGAG

## References

[1] ICTV The ICTV Report Virus Taxonomy: The Classification and Nomenclature of Viruses. https://ictv.global/taxonomy/.

[2] Smithburn K.C., Hughes T.P., Burke A.W., Paul J.H. (1940). A Neurotropic Virus Isolated from the Blood of a Native of Uganda. Am. J. Trop. Med..

[3] Sule W.F., Oluwayelu D.O., Hernández-Triana L.M., Fooks A.R., Venter M., Johnson N. (2018). Epidemiology and Ecology of West Nile Virus in Sub-Saharan Africa. Parasit. Vectors.

[4] Rossi S.L., Ross T.M., Evans J.D. (2010). West Nile Virus. Clin. Lab. Med..

[5] Monini M., Falcone E., Busani L., Romi R., Ruggeri F.M. (2010). West Nile Virus: Characteristics of an African Virus Adapting to the Third Millennium World. Open Virol. J..

[6] Bowen R.A., Nemeth N.M. (2007). Experimental Infections with West Nile Virus. Curr. Opin. Infect. Dis..

[7] Habarugira G., Suen W.W., Hobson-Peters J., Hall R.A., Bielefeldt-Ohmann H. (2020). West Nile Virus: An Update on Pathobiology, Epidemiology, Diagnostics, Control and “One Health” Implications. Pathogens.

[8] Rizzoli A., Jimenez-Clavero M.A., Barzon L., Cordioli P., Figuerola J., Koraka P., Martina B., Moreno A., Nowotny N., Pardigon N. (2015). The Challenge of West Nile Virus in Europe: Knowledge Gaps and Research Priorities. Eurosurveillance.

[9] Zeller H.G., Schuffenecker I. (2004). West Nile Virus: An Overview of Its Spread in Europe and the Mediterranean Basin in Contrast to Its Spread in the Americas. Eur. J. Clin. Microbiol. Infect. Dis..

[10] Hernández-Triana L.M., Jeffries C.L., Mansfield K.L., Carnell G., Fooks A.R., Johnson N. (2014). Emergence of West Nile Virus Lineage 2 in Europe: A Review on the Introduction and Spread of a Mosquito-Borne Disease. Front. Public Health.

[11] Chancey C., Grinev A., Volkova E., Rios M. (2015). The Global Ecology and Epidemiology of West Nile Virus. Biomed Res. Int..

[12] European Centre for Disease Prevention and Control Surveillance of West Nile Virus Infections in Humans and Animals in Europe. https://www.ecdc.europa.eu/en/infectious-disease-topics/west-nile-virus-infection/surveillance-and-disease-data/monthly-updates.

[13] Centers for Disease Control and Prevention (CDC) West Nile Virus. https://www.cdc.gov/west-nile-virus/index.html.

[14] L’vov D.K., Savchenko S.T., Alekseev V.V., Lipnitsky A.V., Pashanina T.P. (2008). Epidemiological Situation and Prognostication of the West Nile Fever Morbidity in the Territory of the Russian Federation. Probl. Part. Danger. Infect..

[15] Putintseva E.V., Udovichenko S.K., Nikitin D.N., Boroday N.V., Antonov A.S., Toporkov A.V. (2024). West Nile Fever: Analysis of the Epidemiological Situation in the Russian Federation in 2023, Forecast for 2024. Probl. Part. Danger. Infect..

[16] Putintseva E.V., Udovichenko S.K., Nikitin D.N., Boroday N.V., Koloskova A.Y., Antonov A.S., Bondareva O.S., Toporkov A.V. (2025). West Nile Fever in the Russian Federation in 2024, Forecast for 2025. Probl. Part. Danger. Infect..

[17] The Federal Service for Surveillance on Consumer Rights Protection and Human Welfare (Rospotrebnadzor) Continues to Monitor the West Nile Fever Situation. https://rospotrebnadzor.ru/region/rss/rss.php?ELEMENT_ID=28278.

[18] Sampathkumar P. (2003). West Nile Virus: Epidemiology, Clinical Presentation, Diagnosis, and Prevention. Mayo Clin. Proc..

[19] MacIntyre C., Lourens C., Mendes A., de Villiers M., Avenant T., du Plessis N.M., Leendertz F.H., Venter M. (2023). West Nile Virus, an Underdiagnosed Cause of Acute Fever of Unknown Origin and Neurological Disease among Hospitalized Patients in South Africa. Viruses.

[20] Klingelhöfer D., Braun M., Kramer I.M., Reuss F., Müller R., Groneberg D.A., Brüggmann D. (2023). A Virus Becomes a Global Concern: Research Activities on West-Nile Virus. Emerg. Microbes Infect..

[21] García-Carrasco J.-M., Muñoz A.-R., Olivero J., Segura M., Real R. (2023). An African West Nile Virus Risk Map for Travellers and Clinicians. Travel Med. Infect. Dis..

[22] Karim S.-U., Bai F. (2023). Introduction to West Nile Virus. Methods in Molecular Biology.

[23] Mukhopadhyay S., Kim B.-S., Chipman P.R., Rossmann M.G., Kuhn R.J. (2003). Structure of West Nile Virus. Science.

[24] Heinz F.X., Stiasny K. (2012). Flaviviruses and Their Antigenic Structure. J. Clin. Virol..

[25] Zhang Y. (2003). Structures of Immature Flavivirus Particles. EMBO J..

[26] Zhang X., Jia R., Shen H., Wang M., Yin Z., Cheng A. (2017). Structures and Functions of the Envelope Glycoprotein in Flavivirus Infections. Viruses.

[27] Brinton M.A. (2001). Host Factors Involved in West Nile Virus Replication. Ann. N. Y. Acad. Sci..

[28] Lieberman M.M., Clements D.E., Ogata S., Wang G., Corpuz G., Wong T., Martyak T., Gilson L., Coller B.-A., Leung J. (2007). Preparation and Immunogenic Properties of a Recombinant West Nile Subunit Vaccine. Vaccine.

[29] Gould L.H., Sui J., Foellmer H., Oliphant T., Wang T., Ledizet M., Murakami A., Noonan K., Lambeth C., Kar K. (2005). Protective and Therapeutic Capacity of Human Single-Chain Fv-Fc Fusion Proteins against West Nile Virus. J. Virol..

[30] Beasley D.W.C., Barrett A.D.T. (2002). Identification of Neutralizing Epitopes within Structural Domain III of the West Nile Virus Envelope Protein. J. Virol..

[31] Kaaijk P., Luytjes W. (2018). Are We Prepared for Emerging Flaviviruses in Europe? Challenges for Vaccination. Hum. Vaccin. Immunother..

[32] Gould C.V., Staples J.E., Huang C.Y.-H., Brault A.C., Nett R.J. (2023). Combating West Nile Virus Disease—Time to Revisit Vaccination. N. Engl. J. Med..

[33] Monath T.P., Liu J., Kanesa-Thasan N., Myers G.A., Nichols R., Deary A., McCarthy K., Johnson C., Ermak T., Shin S. (2006). A Live, Attenuated Recombinant West Nile Virus Vaccine. Proc. Natl. Acad. Sci. USA.

[34] Ozharovskaia T., Popova O., Zubkova O., Vavilova I., Pochtovyy A., Shcheblyakov D., Gushchin V., Logunov D.y., Gintsburg A. (2023). Development and Characterization of a Vector System Based on the Simian Adenovirus Type 25. Bull. Russ. State Med. Univ..

[35] Kanegae Y., Makimura M., Saito I. (1994). A Simple and Efficient Method for Purification of Infectious Recombinant Adenovirus. Jpn. J. Med. Sci. Biol..

[36] Maizel J.V., White D.O., Scharff M.D. (1968). The Polypeptides of Adenovirus. I. Evidence for Multiple Protein Components in the Virion and a Comparison of Types 2, 7A, and 12. Virology.

[37] (2009). Principles of Good Laboratory Practice.

[38] Setoh Y.X., Prow N.A., Hobson-Peters J., Lobigs M., Young P.R., Khromykh A.A., Hall R.A. (2012). Identification of Residues in West Nile Virus Pre-Membrane Protein That Influence Viral Particle Secretion and Virulence. J. Gen. Virol..

[39] Guo L.-P., Huo H., Wang X.-L., Bu Z.-G., Hua R.-H. (2014). Generation and Characterization of a Monoclonal Antibody against PrM Protein of West Nile Virus. Monoclon. Antib. Immunodiagn. Immunother..

[40] Kovalev S.Y., Mukhacheva T.A., Kokorev V.S., Belyaeva I.V. (2012). Tick-Borne Encephalitis Virus: Reference Strain Sofjin and Problem of Its Authenticity. Virus Genes.

[41] Tukhvatulin A.I., Dolzhikova I.V., Shcheblyakov D.V., Zubkova O.V., Dzharullaeva A.S., Kovyrshina A.V., Lubenets N.L., Grousova D.M., Erokhova A.S., Botikov A.G. (2021). An Open, Non-Randomised, Phase 1/2 Trial on the Safety, Tolerability, and Immunogenicity of Single-Dose Vaccine “Sputnik Light” for Prevention of Coronavirus Infection in Healthy Adults. Lancet Reg. Health-Eur..

[42] Logunov D.Y., Dolzhikova I.V., Shcheblyakov D.V., Tukhvatulin A.I., Zubkova O.V., Dzharullaeva A.S., Kovyrshina A.V., Lubenets N.L., Grousova D.M., Erokhova A.S. (2021). Safety and Efficacy of an RAd26 and RAd5 Vector-Based Heterologous Prime-Boost COVID-19 Vaccine: An Interim Analysis of a Randomised Controlled Phase 3 Trial in Russia. Lancet.

[43] Voysey M., Clemens S.A.C., Madhi S.A., Weckx L.Y., Folegatti P.M., Aley P.K., Angus B., Baillie V.L., Barnabas S.L., Bhorat Q.E. (2021). Safety and Efficacy of the ChAdOx1 NCoV-19 Vaccine (AZD1222) against SARS-CoV-2: An Interim Analysis of Four Randomised Controlled Trials in Brazil, South Africa, and the UK. Lancet.

[44] Lu L., Zhang F., Oude Munnink B.B., Munger E., Sikkema R.S., Pappa S., Tsioka K., Sinigaglia A., Dal Molin E., Shih B.B. (2024). West Nile Virus Spread in Europe: Phylogeographic Pattern Analysis and Key Drivers. PLoS Pathog..

[45] Rey F.A., Stiasny K., Vaney M., Dellarole M., Heinz F.X. (2018). The Bright and the Dark Side of Human Antibody Responses to Flaviviruses: Lessons for Vaccine Design. EMBO Rep..

[46] Katzelnick L.C., Narvaez C., Arguello S., Lopez Mercado B., Collado D., Ampie O., Elizondo D., Miranda T., Bustos Carillo F., Mercado J.C. (2020). Zika Virus Infection Enhances Future Risk of Severe Dengue Disease. Science.

[47] Weiß R., Issmail L., Rockstroh A., Grunwald T., Fertey J., Ulbert S. (2023). Immunization with Different Recombinant West Nile Virus Envelope Proteins Induces Varying Levels of Serological Cross-Reactivity and Protection from Infection. Front. Cell. Infect. Microbiol..

[48] Bardina S.V., Bunduc P., Tripathi S., Duehr J., Frere J.J., Brown J.A., Nachbagauer R., Foster G.A., Krysztof D., Tortorella D. (2017). Enhancement of Zika Virus Pathogenesis by Preexisting Antiflavivirus Immunity. Science.

[49] Richner J.M., Himansu S., Dowd K.A., Butler S.L., Salazar V., Fox J.M., Julander J.G., Tang W.W., Shresta S., Pierson T.C. (2017). Modified MRNA Vaccines Protect against Zika Virus Infection. Cell.

[50] Yang M., Lai H., Sun H., Chen Q. (2017). Virus-like Particles That Display Zika Virus Envelope Protein Domain III Induce Potent Neutralizing Immune Responses in Mice. Sci. Rep..

[51] Treangen T.J., Abraham A.-L., Touchon M., Rocha E.P.C. (2009). Genesis, Effects and Fates of Repeats in Prokaryotic Genomes. FEMS Microbiol. Rev..

[52] Bello M.B., Alsaadi A., Naeem A., Almahboub S.A., Bosaeed M., Aljedani S.S. (2024). Development of Nucleic Acid-Based Vaccines against Dengue and Other Mosquito-Borne Flaviviruses: The Past, Present, and Future. Front. Immunol..

[53] Liu X., Qu L., Ye X., Yi C., Zheng X., Hao M., Su W., Yao Z., Chen P., Zhang S. (2018). Incorporation of NS1 and PrM/M Are Important to Confer Effective Protection of Adenovirus-Vectored Zika Virus Vaccine Carrying E Protein. npj Vaccines.

[54] Chang S., Guo X., Han X., Li M., Zhai C., Bing J., Jin L., Jiang Y., Li J., Wang T. (2025). Humoral and Cellular Immune Response to a Single Dose of a Novel Bivalent Recombinant Adenovirus-Vector Vaccine against West Nile Virus and Chikungunya Virus in Mice. Virol. J..

[55] Chung K.M., Liszewski M.K., Nybakken G., Davis A.E., Townsend R.R., Fremont D.H., Atkinson J.P., Diamond M.S. (2006). West Nile Virus Nonstructural Protein NS1 Inhibits Complement Activation by Binding the Regulatory Protein Factor H. Proc. Natl. Acad. Sci. USA.

[56] Samuel M.A., Diamond M.S. (2006). Pathogenesis of West Nile Virus Infection: A Balance between Virulence, Innate and Adaptive Immunity, and Viral Evasion. J. Virol..

[57] de Freitas Costa E., Streng K., Avelino de Souza Santos M., Counotte M.J. (2024). The Effect of Temperature on the Boundary Conditions of West Nile Virus Circulation in Europe. PLoS Negl. Trop. Dis..

[58] Rudolf I., Betášová L., Blažejová H., Venclíková K., Straková P., Šebesta O., Mendel J., Bakonyi T., Schaffner F., Nowotny N. (2017). West Nile Virus in Overwintering Mosquitoes, Central Europe. Parasit. Vectors.

[59] Kampen H., Tews B.A., Werner D. (2021). First Evidence of West Nile Virus Overwintering in Mosquitoes in Germany. Viruses.

[60] Blom R., Schrama M.J.J., Spitzen J., Weller B.F.M., van der Linden A., Sikkema R.S., Koopmans M.P.G., Koenraadt C.J.M. (2023). Arbovirus Persistence in North-Western Europe: Are Mosquitoes the Only Overwintering Pathway?. One Health.

[61] Chu J.-H.J., Chiang C.-C.S., Ng M.-L. (2007). Immunization of Flavivirus West Nile Recombinant Envelope Domain III Protein Induced Specific Immune Response and Protection against West Nile Virus Infection. J. Immunol..

[62] Martina B.E., Koraka P., van den Doel P., van Amerongen G., Rimmelzwaan G.F., Osterhaus A.D.M.E. (2008). Immunization with West Nile Virus Envelope Domain III Protects Mice against Lethal Infection with Homologous and Heterologous Virus. Vaccine.

