# Supplementary figures and images for: Adenoviral Vectors Expressing Optimized preM/E Genes of WNV Deliver Long-Term Protection Against Lethal West Nile Virus Challenge
Source: Vaccines (Basel). 2025 Nov 21;13(12):1177. doi: 10.3390/vaccines13121177 (PMC12737445; doi:10.3390/vaccines13121177)

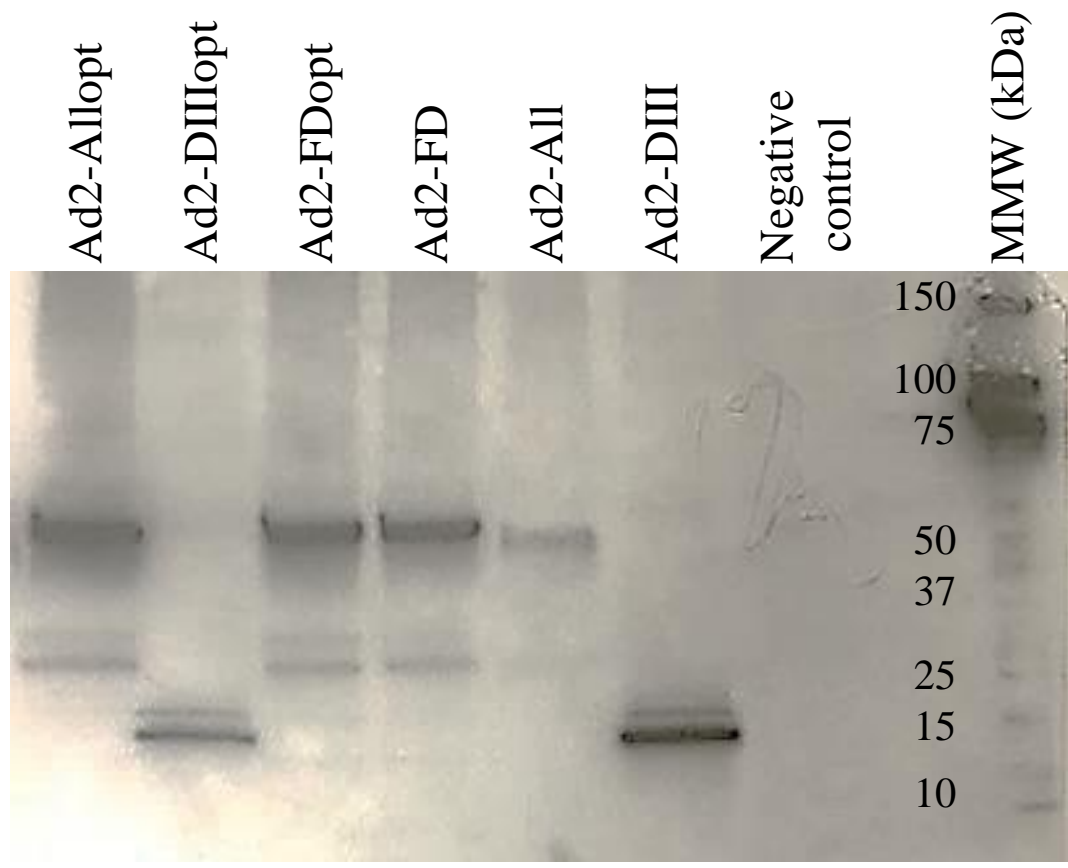

Supplement: Supplementary file 1 [file vaccines-13-01177-s001.zip › vaccines-3981427-supplementary.pdf]
